# Short-Time Intermittent Preexposure of Living Human Donors to Hyperoxia Improves Renal Function in Early Posttransplant Period: A Double-Blind Randomized Clinical Trial

**DOI:** 10.1155/2011/204843

**Published:** 2011-04-07

**Authors:** Kamran Montazeri, Mohammadali Vakily, Azim Honarmand, Parviz Kashefi, Mohammadreza Safavi, Shahram Taheri, Bahram Rasoulian

**Affiliations:** ^1^Anesthesiology and Critical Care Research Center, Isfahan University of Medical Sciences, P.O. Box 8174675731, Isfahan 81744, Iran; ^2^Isfahan Kidney Diseases Research Center (IKRC) and Internal Medicine Department, Alzahra Hospital, Isfahan University of Medical Sciences, P.O. Box 8174675731, Isfahan 81744, Iran; ^3^Research Center and Department of Physiology, Lorestan University of Medical Sciences, P.O. Box 6814617767, Khorramabad, Iran

## Abstract

The purpose of this human study was to investigate the effect of oxygen pretreatment in living kidney donors on early renal function of transplanted kidney. Sixty living kidney donor individuals were assigned to receive either 8–10 L/min oxygen (Group I) by a non-rebreather mask with reservoir bag intermittently for one hour at four times (20, 16, 12, and 1 hours before transplantation) or air (Group II). After kidney transplantation, urine output, blood urea nitrogen (BUN), serum creatinine, need to additional diuretics (NTADs) in the first 24 hours after transplantation, delayed graft function (DGF), the creatinine clearance (CrCL) on 10th day, and duration of hospital stay from the first posttransplant day till normalization of renal function was recorded and compared in two groups. Mean CrCL in posttransplant day 10, NTAD after 24 hours of transplantation, and urine output during 6 hours after operation were significantly better in Group I compared with Group II (*P* < .05). Also, DGF during the first week after operation and duration of hospital stay was less in Group I compared with Group II. Intermittent exposure of human living kidney donor to hyperoxic environment may improve renal function following kidney transplantation.

## 1. Introduction

Preservation of renal function is an essential purpose in renal transplantation and in many other vascular and urological procedures where renal functional impairment follows ischemia-reperfusion (I-R) injury [1]. Ischemic injury occurs when the blood supply to a tissue is interrupted, but paradoxically more severe tissue injury arises when blood flow is restored on reperfusion [2]. Renal warm I-R injury happens during kidney transplantation and has a major impact on morbidity [3], on cost increase [4], and on prognosis [5, 6]. 

Delayed graft function (DGF) was defined as hemodialysis requirement during the first week after renal transplantation. Reperfusion injury is a risk factor for development of DGF after transplantation [7]. 

Therefore, protective maneuvers before transplantation could obviously profit the fate of the organ. Murry et al. [8] in 1986 demonstrated that the application of short-time episodes of ischemia and reperfusion to the dog's myocardium led to the development of tolerance to subsequent more prolonged ischemia and reperfusion and implement the term “ischemic preconditioning” (IPC) to express this endogenous inducible protection. This phenomenon has been primarily investigated and characterized in the heart [9, 10], but it has also been explained in the liver [11], the small intestine [12], lung [13], brain [14], and kidney [15]. 

Inducing brief episodes of ischemia and reperfusion in human appears fairly aggressive and not satisfactory by many surgeons [16]. Accordingly finding safer methods of preconditioning against IR injury is crucial. 

Amongst the most practical methods for induction of ischemic tolerance in tissues is short-period exposure to hyperoxia which has no significant adverse effects and occasionally appears to be beneficial (e.g., by maximizing arterial oxygen saturation). In animal models, the protective effects of oxygen pretreatment on subsequent ischemia-reperfusion injury have been confirmed in heart [17, 18], brain [19], spinal cord [20], and finally kidney [21, 22]. 

Wahhabaghai and colleagues [22] showed that repeated exposure to hyperoxic (≥95% O_2_) environment can decrease rat's renal ischemia-reperfusion damage.

Since free radical formation is the main cause of IR injury [1], the mechanism of hyperoxia-induced preconditioning against IR injury appears to be induction of endogenous defense strategies against free radicals by low grade oxidative stress resulting from short period of exposure to hyperoxia [18]. 

To the best of our knowledge, there is no human study which indicates this effect of oxygen pretreatment on renal function of transplanted kidney. Therefore, the present study was undertaken to determine whether brief exposure to the hyperoxia in living donors could improve renal function measured throughout 10 days after kidney transplantation.

## 2. Material and Methods

From October 2007 to December 2009, sixty ASA (American society of Anesthesiology) III patients with end-stage renal disease who had undergone first kidney transplant from a living donor, aged 18–65 years old, with no history of previous transplantation, human immunodeficiency, and hepatitis B and C virus infection were recruited to participate in this randomized, double blind clinical trial study.

The kidney donors were 18–55 years old healthy (ASA I) individuals who were evaluated for kidney donation by corresponding nephrologists and their own recipients were WBC cross match negative.

The study protocol was approved by the medical ethics research committee of our university, and written informed consent was obtained from each kidney donor. 

Recipients with new onset of any major complications (myocardial infarction, stroke, hemorrhagic shock, etc.) after transplantation, female donors to male recipients, and donors who were noncompliant with study protocol and received maintenance anesthesia other than isoflurane were excluded from the study. 

At enrolment, living kidney donors were assigned by a computer-generated list of random numbers to receive either 8–10 L/min oxygen (Group I) by a non-rebreather mask with reservoir bag intermittently for one hour at four times (20, 16, 12, and 1 hours before transplantation) or air (Group II).

 In the first five donors in each group, an arterial blood gas (ABG) test was done with permission of the participants for measuring the oxygen arterial partial pressure (P_a_O_2_) and assessing the effectiveness of oxygen administration.

Recipients and their medical team were blinded to the study group assignments. Before transplant operation, all recipients received 500 mg IV methylprednisolone, 6–8 mg/kg cyclosporine A, and 1 gram mycophenolate mofetil.

Data concerned recipients (age, weight, gender, primary renal disease, time on dialysis, urinary output status (oliguric or anuric), type of dialysis, serology to hepatitis C and B virus, and last panel-reactive antibodies), and donors (age, gender, and serology to hepatitis C and B virus) were recorded in both groups.

No anesthetic premedication was given to the recipients and donors. Peripheral oxygen saturation (SpO_2_), electrocardiogram, noninvasive arterial blood pressure, and heart rate were recorded in the operating room. After 3–5 minutes preoxygenation with 100% oxygen, anesthesia was induced with thiopental 5 mg/kg and fentanyl 3 *μ*g/kg followed by atracurium 0.6 mg/kg to facilitate tracheal intubation. Anaesthesia was maintained with isoflurane 1–1.2% and nitrous oxide 50% in oxygen, and patients were maintained under controlled ventilation to achieve end-tidal CO_2_ values of 32–35 mmHg. 

In recipients, fentanyl 1 *μ*g/kg was given as rescue medication when insufficient analgesia was noted (defined as a heart rate or systolic arterial blood pressure that exceeded baseline values by 20%). All recipients received normal saline 0.9% 70 mL/kg and furosemide 3 mg/kg before completion of vascular anastomosis. Residual neuromuscular blockade was reversed by neostigmine 40 *μ*g/kg and atropine 20 *μ*g/ kg at the end of surgery.

After emergence from anaesthesia, patients were cared in the postanaesthetic care unit (PACU). They received supplemental O_2_ at a rate of 2–4 L/min with nasal prongs and were transferred to the kidney transplant intensive care unit after stabilization. 

In donors, induction and maintenance of anesthesia were similar and so for recipient patients. In donors, patients received 30 mL/kg Ringer until just before open nephrectomy. After nephrectomy, the kidney was prepared in University of Wisconsin (UW) solution at 4°C temperature and immediately transplanted to recipients.

After kidney transplantation, urine output was monitored closely for volume hourly for the first 24 hours and recorded for statistical analysis during 1, 6, and 24 hours. If the patients had urine output volume less than 200 mL per hour in spite of adequate fluid therapy, additional diuretics (furosemide 10 mg per hour) were administered. Also, blood urea nitrogen (BUN) and serum creatinine (sCr), serum sodium and potassium were tested and recorded daily for 10 days. 

Urine sodium and creatinine were measured, and the fractional excretion of sodium (FENa) was calculated on the days 2 and 4 after kidney transplant. Any patients' DGF was recorded.

The creatinine clearance (CrCL) on 10th day and duration of hospital stay were also recorded in two groups. 

Duration of hospital stay was defined as days required for renal functions become stabilized after transplantation day. Acute rejection (AR) was defined based on clinical and/or paraclinical data (biopsy or renal artery color Doppler sonography or nuclear scan of transplanted kidney). If any patient had AR, it was recorded and treated according to the standard protocols. Maintenance of immunosuppression was cyclosporine A 3-4 mg/kg q12 h and adjusted to maintain trough blood level 150–300 ng/mL, mycophenolate mofetil 500–1000 mg q12 h, and prednisolone 1 mg/kg. 

All data were analyzed with SPSS 16 (SPSS Inc, Chicago, IL, USA). Patient characteristics were described as means ± SDs for continuous variables and frequency for categorical variables. Student's *t-*test for unpaired data, or chi-square analysis, was used as appropriate, to assess differences between two groups. A *P* value of less than .05 was considered statistically significant.

## 3. Results

**Figure 1:**
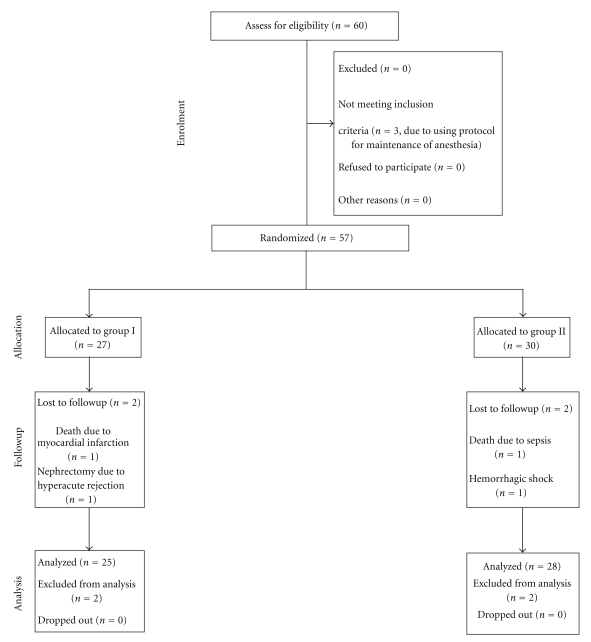
Flow diagram of the progress through the phases of the randomised trial. Group I: living kidney donor patients under open nephrectomy received high-flow oxygen; Group II: living kidney donor patients under open nephrectomy received air.

Sixty patients completed the study criteria for randomization. Seven patients dropped out of the study (see CONSORT Statement, Figure 1).

There were no significant differences between the two study groups with respect to the data concerning recipients (age, gender, duration of CRF, duration of hemodialysis, the incidence of anuria-oliguria, and causes of chronic renal failure) and donors (age and gender) (Table 1). Mean (± SD) partial pressure of oxygen in arterial blood (P_a_O_2_) in the first five donor participants from group I and group II was 303 ± 7.8 and 85 ± 3.7 mmHg, respectively.

Need to additional diuretic in the first 24 hours after the operation was significantly less in Group I compared to Group II (*P* < .05) (Table 2).

Urine output during 6 hours after operation was significantly more in Group I compared to Group II (*P* < .05) (Table 2). The incidence of DGF was more in Group II (four patients, i.e., 14.3%) compared to Group I (only one patient i.e., 4%), but this difference was not significant statistically (Table 2).

**Figure 2:**
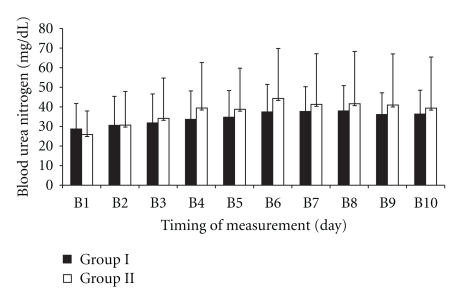
Comparison of blood urea nitrogen (BUN) measured daily for ten days after kidney transplantation in two groups. Dates are presented as mean ± SD. Group I: living kidney donor patients under open nephrectomy received high-flow oxygen; Group II: living kidney donor patients under open nephrectomy received air. B: blood urea nitrogen. BUN was less in Group I compared with Group II on postoperative days of 3–10, but it was not statistically significant.

**Figure 3:**
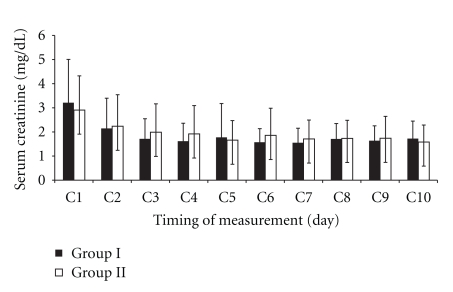
Comparison of serum creatinine measured daily for ten days after kidney transplantation in two groups. Dates are presented as mean ± SD. Group I: living kidney donor patients under open nephrectomy received high-flow oxygen; Group II: living kidney donor patients under open nephrectomy received air. C: serum creatinine. There was no significant difference between the two groups.

There was no significant difference in FENa_2_ and FENa_4_ between the two groups (Table 2). Mean creatinine clearance in posttransplant day 10 was significantly less in Group II compared with Group I (*P* < .05) (Table 2). There was no significant difference in BUN or sCr measured daily for ten days after kidney transplantation among two groups (Figures 2 and 3).

Mean BUN and sCr on posttransplant day 10 was less in Group I compared with Group II, but it was not significant statistically (Table 2).

Mean duration of hospital stay from the first posttransplant day till normalization of renal function was about 5.2 days lower in Group I compared to Group II, and this difference was statistically significant (*P* < .05) (Table 2).

There was not any statistically significant difference between the rates of postoperative complications among the two groups (Table 2).

## 4. Discussion

The major finding of our study was that the intermittent exposure of living human kidney donors to the hyperoxia improves early renal function measured in the first ten days after kidney transplantation. 

Mean serum BUN and creatinine throughout posttransplant day 1 to 10, creatinine clearance in day 10, need to additional diuretics in the first 24 hours after operation, urine output during 6 hours after operation, and also duration of hospital stay were significantly better in Group I who were pretreated with hyperoxia compared with Group II who had no such exposure.

As our study showed that the incidence of DGF was insignificantly more in Group II (14.3%) compared with Group I (4%). Generally, the great majority of kidney transplants are carried out using kidneys from standard criteria donors with moderate DGF rates of 21–31% [23, 24].

A number of factors have been documented to impact short-term graft survival. These consist of delayed allograft function, HLA antibodies, type of donor kidney, donor illness, medical center factors, and other factors.

Allograft injury participates an important role in both short- and long-term graft function, as well as in the induction of renal allograft rejection [25]. Such injury possibly is induced by different events, including brain death, cold ischemia time, ischemia and/or reperfusion, and infection.

Ischemia and/or reperfusion injury is supposed to be a critical risk factor for both early delayed graft function and late allograft dysfunction. 

The major cause of delayed graft function is postischemic acute tubular necrosis (ATN) [26]. A number of authors are of the belief that duration of the vascular anastomosis more than 35 minutes may be a factor to the development of ATN [27]. 

In Szostek and colleagues [28] study, the value of effective cooling of the kidney during the vascular anastomosis in preventing development of ATN was documented. In univariate analysis of several factors that could be a factor to the development of ATN, it was shown that donor hypotension, type of kidney storage, and temperature rise during the anastomosis had significant effect [28].

The conditions surrounding organ removal, storage, and engraftment possibly will enhance graft immunogenicity [29–31]. Such factors comprise the upregulation of major histocompatibility complex (MHC) antigens and triggering of the cytokine-adhesion molecule cascade [32, 33].

Old and very young kidneys have moderately reduced numbers of functioning nephrons and stay alive less well once transplanted. Besides fewer nephrons other factors intrinsic to an older kidney may well impact whole allograft survival [34]. Large sized recipients put a great physiologic demand on moderately “inadequate” numbers of transplanted nephrons possibly will be a factor in the lower graft survival rate [35]. 

Differences in the capacity to produce an efficient immune response versus the allograft as well as variations in primary factors influencing allograft fibrosis signify alloantigen-independent factors that may impinge on graft survival [36]. 

The above-discussed factors are the main cause of graft survival that may influence 14.3% incidence of DGF in the Group II patients. One limitation of our study was that we did not investigate the role of each factor separately. 

The methods used in our study did not allow us to identify the mechanisms by which hyperoxic preconditioning protected the transplanted kidney. We hypothesized that increased oxygen volume dissolved in blood plasma resulted in enhanced oxygen supply to marginally perfused tissue. 

Higher plasma oxygen concentration possibly will be important because capillary blood flow during ischemia can mainly consist of plasma flow [37]. Better oxygen delivery may have led to improvement of energy metabolism in penumbral regions and decreasing their vulnerability to additional metabolic challenges such as peri-infarct depolarizations [38]. 

On the other hand, several other mechanisms implicated in ischemia and reperfusion injury may be influenced by hyperoxia. Ahrens and colleagues [39] found a decrease of infarct size in mice which had been pretreated with hyperoxia several days before focal ischemia. This preconditioning effect might suggest that hyperoxia triggers reactive de novo expression of protective genes taking part in free radicals decay.

Thom et al. [40] and Warner et al. [41] showed that hyperoxia treatment inhibited the function of neutrophil beta-2-integrin, a molecule involved in leukocyte adhesion and reperfusion injury.

For the following reasons, oxygen-free radicals (OFRs) may present a common signal in ischemic tolerance (IT) induction: (1) the majority if not all of published tolerance inducing stimuli are associated with the production of OFR; (2) induction of antioxidant enzymes has been shown for many IT protocols; (3) OFR itself may induce IT [42].

Hyperoxic oxygenation, the exposure of an organism to an environment of relatively pure oxygen, increases physiological OFR production in all organs, including the kidney [43–45].

Rasoulian et al. [21] and Wahhabaghai et al. [22] showed that intermittent pre-exposure to hyperoxic environment can reduce subsequent renal ischemic injury in rats.

The mechanism of advantage of hyperoxemia might involve the vascular element with impaired autoregulation in the area of ischemia. Vasoconstriction caused by a high P_a_O_2_ permits shunting of blood into the infarct from nearby normal brain [46].

Donor hyperoxia, defined as a P_a_O_2_ value more than 150 mmHg, was associated with improved graft survival even independently from the classification of the quality of early postoperative graft function. Donor hyperoxia might induce good early postoperative liver function by improving ATP hepatic content, increasing protein synthesis, and decreasing the proteolytic process in the liver. It was also proposed that donor hyperoxia could be valuable, as it has been demonstrated experimentally that hyperoxic pretreatment attenuates ischemic-reperfusion injury of the heart [18, 47], brain [19, 48], spinal cord [20], liver [49], and kidney [21, 22].

We hypothesize that early hyperoxia treatment may prolong the short-time window for therapeutic interventions, a major management problem in ischemic stroke. Nevertheless, we caution against overinterpretation of our data because we did not study the effect of hyperoxia during reperfusion or in permanent focal ischemia. Future studies should address these issues and recognize the exact mechanisms and pathophysiologic targets of hyperoxia. 

Pure oxygen at atmospheric pressure is nontoxic if given for less than 6 hours, and 80% oxygen could be administered for 24 hours [50] far away from four times of one-hour exposure which was employed in our study. 

The most important complication of short periods of oxygen administration is pulmonary atelectasis [51] and vomiting [51]. Preoxygenation with 80% oxygen is associated with significantly lower rates of atelectasis compared with 100% oxygen [52]. Not only pure oxygen but also 80% oxygen has protective effects on rat heart tissue [18], and this may be correct for other tissues like kidney and keeps on to be studied.

In conclusion, intermittent exposure of human living kidney donors to hyperoxic environment improves renal function in early period following kidney transplantation.

## Figures and Tables

**Table 1:** Preoperative characteristic of the donors and recipients.

Variable	Group I	Group II
	(*n* = 25)	(*n* = 28)
*Donor*		
Age (years)	27.0 ± 4.8	32.0 ± 5.4
Sex (M/F)	25/0	28/0
*Recipient*		
Age (yr)	39.6 ± 11.7	39.0 ± 13.1
Sex (M/F)	20/5	19/9
Anuria/oliguria	2/23	3/25
Duration of CRF (month)	40.0 ± 8	32.0 ± 9
DOH (month)	22.0 ± 4	23.0 ± 6
Cause of CRF		
Glomerulonephritis	2	4
Hypertension	8	6
Diabetes mellitus	5	2
Pyelonephritis	1	1
SLE	1	2
ATN	1	0
Urologic disease	0	2
(VUR, nephrolithiasis, etc.)		
Unknown	0	1
Other	7	10

Dates are presented as mean ± SD or numbers. Group I: living kidney donor patients under open nephrectomy received high flow oxygen; Group II: living kidney donor patients under open nephrectomy received air. CRF: chronic renal failure; DOH: duration of hemodialysis; SLE: systemic lupus erythematosus; ATN: acute tubular necrosis; VUR: vesicoureteral reflux. There was no significant difference between the two groups.

**Table 2:** Postoperative characteristic and complications of recipients.

Variable	Group I (*n* = 25)	Group II (*n* = 28)	*P* value
NTAD			
At first hours AO	0^*α*^	4	.07
At 24 hours AO	2*	10	.017
Urine output (mL)			
At first hours AO	516 ± 443	451 ± 428	.594
At 6 hours AO	4522 ± 1899*	3185 ± 2253	.024
At 24 hours AO	17990 ± 6122	16368 ± 20386	.704
NTH during first week	1	4	.213
FENa_2_ (%)	7.2 ± 4.5	7.2 ± 9.7	.979
FENa_4_ (%)	3.6 ± 2.0	3.3 ± 4.3	.715
CrCL_10_ (mL/min)	61.6 ± 18.6*	49.8 ± 20.0	.033
Creatinine_10_ (mg/dL)	1.8 ± 0.7	1.96 ± 0.9	.522
BUN_10_ (mg/dL)	34 ± 12	38.2 ± 19.7	.352
Hospital stays (days)	13.9 ± 2.7*	19.1 ± 10.1	.025
Complications	4	6	.450
Acute rejection	4	4	
ATN	0	1	
MI	0	1	

Date are presented as mean ± SD or numbers. Group I: living kidney donor patients under open nephrectomy received high-flow oxygen; Group II: living kidney donor patients under open nephrectomy received air. NTAD: need to additional diuretic; AO: after operation; NTH: need to hemodialysis, FENa_2_: fractional excretion of sodium at posttransplant day 2; CrCL_10_: creatinine clearance throughout posttransplant day 1 to 10; BUN_10_: blood urea nitrogen throughout posttransplant day 1 to 10; ATN: acute tubular necrosis; MI: myocardial infarction. *P < .05 versus Group II. ^*α*^ *P* = .07.

## References

[1] Bouchier-Hayes DM, Fitzpatrick JM, Grace PA, Mathie RT (1999). Local consequences of reperfusion in the kidney. *Ischemia-Reperfusion Injury*.

[2] Sumeray MS, Yellon DM, Grace PA, Mathie RT (1999). Ischaemic preconditioning. *Ischaemia-Reperfusion Injury*.

[3] Troppmann C, Gillingham KJ, Benedetti E (1995). Delayed graft function, acute rejection, and outcome after cadaver renal transplantation: a multivariate analysis. *Transplantation*.

[4] Almond PS, Troppmann C, Escobar F, Frey DJ, Matas AJ (1991). Economic impact of delayed graft function. *Transplantation Proceedings*.

[5] Shoskes DA, Cecka JM (1998). Deleterious effects of delayed graft function in cadaveric renal transplant recipients independent of acute rejection. *Transplantation*.

[6] Feldman HI, Gayner R, Berlin JA (1996). Delayed function reduces renal allograft survival independent of acute rejection. *Nephrology Dialysis Transplantation*.

[7] Rowinski W, Chmura A, Kosieradzki M (1999). Delayed kidney function risk score: donor factors versus ischemia/reperfusion injury. *Transplantation Proceedings*.

[8] Murry CE, Jennings RB, Reimer KA (1986). Preconditioning with ischemia: a delay of lethal cell injury in ischemic myocardium. *Circulation*.

[9] Nakano A, Liu GS, Heusch G, Downey JM, Cohen MV (2000). Exogenous nitric oxide can trigger a preconditioned state through a free radical mechanism, but endogenous nitric oxide is not a trigger of classical ischemic preconditioning. *Journal of Molecular and Cellular Cardiology*.

[10] Parratt JR (1994). Protection of the heart by ischaemic preconditioning: mechanisms and possibilities for pharmacological exploitation. *Trends in Pharmacological Sciences*.

[11] Corradini SG, Elisei W, De Marco R (2005). Preharvest donor hyperoxia predicts good early graft function and longer graft survival after liver transplantation. *Liver Transplantation*.

[12] Hotter G, Closa D, Prados M (1996). Intestinal preconditioning is mediated by a transient increase in nitric oxide. *Biochemical and Biophysical Research Communications*.

[13] Neely CF, Keith IM (1995). A adenosine receptor antagonists block ischemia-reperfusion injury of the lung. *American Journal of Physiology*.

[14] Heurteaux C, Lauritzen I, Widmann C, Lazdunski M (1995). Essential role of adenosine, adenosine A1 receptors, and ATP-sensitive K channels in cerebral ischemic preconditioning. *Proceedings of the National Academy of Sciences of the United States of America*.

[15] Torras J, Herrero-Fresneda I, Lloberas N, Riera M, Cruzaoo JM, Grinyo JM (2002). Promising effects of ischemic preconditioning in renal transplantation. *Kidney International*.

[16] Vaage J, Valen G (2003). Preconditioning and cardiac surgery. *Annals of Thoracic Surgery*.

[17] Tähepôld P, Valen G, Starkopf J, Kairane C, Zilmer M, Vaage J (2001). Pretreating rats with hyperoxia attenuates ischemia-reperfusion injury of the heart. *Life Sciences*.

[18] Esmaili Dehaj M, Baharvand B, Rasoulian B (2009). Delayed protective effects of hyperoxia against cardiac arrhythmias and infarction in anesthetized rats. *Journal of Surgical Research*.

[19] Bigdeli MR, Hajizadeh S, Froozandeh M (2008). Normobaric hyperoxia induces ischemic tolerance and upregulation of glutamate transporters in the rat brain and serum TNF-*α* level. *Experimental Neurology*.

[20] Dong H, Xiong L, Zhu Z, Chen S, Hou L, Sakabe T (2002). Preconditioning with hyperbaric oxygen and hyperoxia induces tolerance against spinal cord ischemia in rabbits. *Anesthesiology*.

[21] Rasoulian B, Mohammadhosseniakbari H, Kadkhodaee M (2008). Preconditioning with oxygen attenuates rat renal ischemia-reperfusion injury. *Journal of Surgical Research*.

[22] Wahhabaghai H, Rasoulian B, Esmaili M (2009). Hyperoxia-induced protection against rat’s renal ischemic damage: relation to oxygen exposure time. *Renal Failure*.

[23] Kyllönen LEJ, Salmela KT, Eklund BH (2000). Long-term results of 1047 cadaveric kidney transplantations with special emphasis on initial graft function and rejection. *Transplant International*.

[24] Patel SJ, Duhart BT, Krauss AG (2008). Risk factors and consequences of delayed graft function in deceased donor renal transplant patients receiving antithymocyte globulin induction. *Transplantation*.

[25] Lu CY, Penfield JG, Kielar ML, Vazquez MA, Jeyarajah DR (1999). Hypothesis: is renal allograft rejection initiated by the response to injury sustained during the transplant process. *Kidney International*.

[26] Lim EC, Terasaki PI, Terasaki PI (1991). Early graft function. *Clinical Transplants*.

[27] Halloran P, Aprile M, Farewell V (1988). Factors influencing early renal function in cadaver kidney transplants. A case-control study. *Transplantation*.

[28] Szostek M, Pacholczyk M, Lagiewska B, Danielewicz R, Wałaszwski J, Rwiński W (1996). Effective surface cooling of the kidney during vascular anastomosis decreases the risk of delayed kidney function after transplantation. *Transplant International*.

[29] Peters TG, Shaver TR, Ames JE, Santiago-Delpin EA, Jones KW, Blanton JW (1995). Cold ischemia and outcome in 17,937 cadaveric kidney transplants. *Transplantation*.

[30] Schnuelle P, Lorenz D, Mueller A, Trede M, Van Der Woude FJ (1999). Donor catecholamine use reduces acute allograft rejection and improves graft survival after cadaveric renal transplantation. *Kidney International*.

[31] Bryan CF, Luger AM, Martinez J (2001). Cold ischemia time: an independent predictor of increased hla class I antibody production after rejection of a primary cadaveric renal allograft. *Transplantation*.

[32] Chandraker A, Takada M, Nadeau KC, Peach R, Tilney NL, Sayegh MH (1997). Rapid communication: CD28-B7 blockade in organ dysfunction secondary to cold ischemia/reperfusion injury. *Kidney International*.

[33] Cockcroft DW, Gault MH (1976). Prediction of creatinine clearance from serum creatinine. *Nephron*.

[34] De Fijter JW, Mallat MJK, Doxiadis IIN (2001). Increased immunogenicity and cause of graft loss of old donor kidneys. *Journal of the American Society of Nephrology*.

[35] Brenner BM, Cohen RA, Milford EL (1992). In renal transplantation, one size may not fit all. *Journal of the American Society of Nephrology*.

[36] Moore J, Mcknight AJ, Simmonds MJ (2010). Association of Caveolin-1 gene polymorphism with kidney transplant fibrosis and allograft failure. *Journal of the American Medical Association*.

[37] Theilen H, Schrock H, Kuschinsky W (1994). Gross persistence of capillary plasma perfusion after middle cerebral artery occlusion in the rat brain. *Journal of Cerebral Blood Flow and Metabolism*.

[38] Hossmann KA (1996). Periinfarct depolarizations. *Cerebrovascular and Brain Metabolism Reviews*.

[39] Ahrens M, Wiegand F, Liao W (1998). Cerebral free radicals generated by hyperbaric oxygen induce tolerance against permanent focal ischemia in the rat. *Neuroscience*.

[40] Thom SR, Mendiguren I, Hardy K (1997). Inhibition of human neutrophil *β*-integrin-dependent adherence by hyperbaric O_2_. *American Journal of Physiology*.

[41] Warner DS, Ludwig PS, Pearlstein R, Brinkhous AD (1995). Halothane reduces focal ischemic injury in the rat when brain temperature is controlled. *Anesthesiology*.

[42] Schumann P, Prass K, Wiegand F, Ito U, Fieschi C, Orzi F (1999). Oxygen free radicals and ischaemic preconditioning in the brain: preliminary data and a hypothesis. *Maturation Phenomenon in Cerebral Ischemia III*.

[43] Paller MS, Hoidal JR, Ferris TF (1984). Oxygen free radicals in ischemic acute renal failure in the rat. *Journal of Clinical Investigation*.

[44] Kaneda T, Ku K, Inoue T, Onoe M, Oku H (2001). Postischemic reperfusion injury can be attenuated by oxygen tension control. *Japanese Circulation Journal*.

[45] Zwemer CF, Shoemaker JL, Hazard SW, Davis RE, Bartoletti AG, Phillips CL (2000). Hyperoxic reperfusion exacerbates postischemic renal dysfunction. *Surgery*.

[46] Nakajima S, Meyer JS, Amano T (1983). Cerebral vasomotor responsiveness during 100% oxygen inhalation in cerebral ischemia. *Archives of Neurology*.

[47] Baharvand B, Dehaj ME, Foadaddini M (2010). Delayed cardioprotective effects of hyperoxia preconditioning prolonged by intermittent exposure. *Journal of Surgical Research*.

[48] Bigdeli MR, Rasoulian B, Meratan AA (2009). In vivo normobaric hyperoxia preconditioning induces different degrees of antioxidant enzymes activities in rat brain tissue. *European Journal of Pharmacology*.

[49] Yu SY, Chiu JH, Yang SD (2005). Preconditioned hyperbaric oxygenation protects the liver against ischemia-reperfusion injury in rats. *Journal of Surgical Research*.

[50] Tinits P (1983). Oxygen therapy and oxygen toxicity. *Annals of Emergency Medicine*.

[51] Kabon B, Kurz A (2006). Optimal perioperative oxygen administration. *Current Opinion in Anaesthesiology*.

[52] Edmark L, Enlund M, Kostova-Aherdan K (2001). Atelectasis formation and apnoea tolerance after preoxygenation with 100%, 80%, or 60% oxygen. *Anesthesiology*.

